# Klippel-Feil syndrom: a duplicated thumb

**DOI:** 10.11604/pamj.2018.31.42.16327

**Published:** 2018-09-20

**Authors:** Aryé Weinberg, Andreas Eberhard Albers

**Affiliations:** 1 Prosper-Hospital, Department of Otorhinolaryngology, Head and Neck Surgery, Recklinghausen, Germany; 2 Department of Otorhinolaryngology, Head and Neck Surgery, Berlin Institute of Health, Charité Universitätsmedizin Berlin, Campus Benjamin Franklin, Berlin, Germany

**Keywords:** Klippel-Feil syndrom, duplicated thumb, skeletal disease

## Image in medicine

A 71-year-old woman was admitted to our hospital because of a vertigo. At clinical examination a duplicated right thumb was noticed. The Patient revealed that she was suffering from Klippel-Feil Syndrom (KFS). KFS is a rare skeletal disease where a mutation of the GDF6 and GDF3 genes can be found. It is characterized by congenital fusion of any of the 7 cervical vertebrae. There can be associations with other malformations such as congenital elevation of the scapula (Sprengel’s deformity), spina bifida, scoliosis, cleft palate, malformations of the heart, head, face, arms, legs and fingers. Because of these heterogeneous medical associated conditions treatment for KFS is symptomatic and can include surgery.

**Figure 1:**
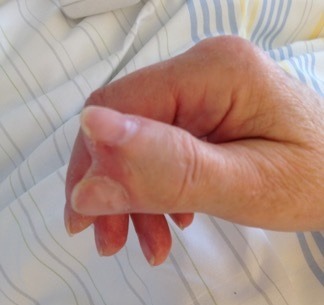
Dublicated thumb

